# Pharmacotherapy for Alcohol Craving Reduction: Efficacy of Short-Term Treatments in Alcohol Use Disorder

**DOI:** 10.3390/medicines13010007

**Published:** 2026-02-14

**Authors:** Matheus Cheibub David Marin, Maria Olivia Pozzolo Pedro, Giuliana Perrotte, João Mauricio Castaldelli-Maia

**Affiliations:** 1 Perdizes Institute (IPer), Clinics Hospital of the Medical School (HCFMUSP), University of São Paulo, São Paulo 05021-001, Brazil; dr.matheus@gmail.com; 2 Department of Psychiatry, Medical School, University of São Paulo, São Paulo 05403-903, Brazil; maria.oliviapozzolo@gmail.com (M.O.P.P.); giuliana.perrotte@hc.fm.usp.br (G.P.)

**Keywords:** alcohol use disorder, craving, pharmacotherapy, short-term treatment, substance dependence, cue-induced craving

## Abstract

Background: Alcohol Use Disorder (AUD) is a major contributor to global morbidity, mortality, and socioeconomic burden. Cravings, defined as intense urges to consume alcohol, play a central role in relapse and are recognized as a diagnostic criterion in DSM-5. Pharmacological strategies targeting cravings may offer immediate or short-term relief, complementing existing long-term approaches. However, evidence on short-term (up to approximately three months) anti-craving interventions remains fragmented. Objective: To systematically review randomized, double-blind, placebo-controlled trials (RCTs) assessing the short-term effects of pharmacological treatments on cue-induced alcohol cravings. Methods: A systematic search was conducted in PubMed and PsycINFO using terms related to alcohol, craving, and randomized controlled designs. Eligibility included clinical trials on alcohol-dependent participants that evaluated craving as an outcome. Exclusion criteria encompassed non-clinical studies, non-pharmacological interventions, animal studies, single-blind trials, and studies with psychiatric comorbidities. Study quality was appraised using Cochrane and Joanna Briggs Institute tools. Results: From 442 studies screened, 26 RCTs fulfilled the inclusion criteria. In total, 1097 participants were enrolled across the trials (range = 16–125 per study; mean = 44), predominantly male outpatients aged 15–65 years. Craving was assessed primarily with the Visual Analog Scale and Alcohol Urge Questionnaire. Intervention duration ranged from 1 to 98 days. Naltrexone consistently reduced cue-induced craving across four trials, with additional benefit observed when combined with ondansetron. Varenicline and acamprosate also demonstrated reductions in craving and drinking. Memantine showed efficacy in craving reduction but was not assessed for abstinence. Topiramate was effective, whereas gabapentin showed limited short-term benefit. Other agents (e.g., citalopram, oxytocin, ondansetron, quetiapine) yielded mixed findings, often limited to single studies. Overall, 58% of trials reported positive anti-craving effects, 23% no difference, and 8% increased craving versus placebo. However, these findings should be interpreted in light of important methodological limitations, including small sample sizes and heterogeneous experimental paradigms. Conclusions: This review suggests that naltrexone and varenicline appear to be the most consistently supported short-term pharmacotherapies for alcohol craving within the available evidence, with promising but less consistent findings for memantine, acamprosate, and topiramate. These results highlight potential candidates for immediate craving management in AUD, while underscoring the need for larger and longer-term trials to confirm their efficacy and safety.

## 1. Introduction

The treatment of substance use disorder remains one of the significant challenges of medical practice. Despite the years of research, few effective treatments are available, and unfortunately, no current treatment guarantees complete effectiveness [1,2]. This is no different for Alcohol Use Disorder (AUD), where specific medications for treatment are available but remain limited in number [3]. AUD is a globally prevalent condition that significantly impacts not only individual health, but also societal and economic well-being. It contributes to an increase in the number of individuals requiring medical care, resulting in substantial healthcare costs and reduced workforce productivity [4].

According to data from the World Health Organization (WHO), alcohol misuse contributes to three million deaths annually, accounting for 5.3% of total global fatalities. Additionally, AUD is a leading factor in over 200 health conditions and injuries. Approximately 5.1% of the worldwide disease burden and injuries are attributed to alcohol consumption, measured in terms of Disability-Adjusted Life Years (DALYs) [5]. Alcohol consumption is associated with death and disability relatively early in life. Among those aged 20–39, approximately 13.5% of all deaths are attributable to alcohol. There is a causal relationship between harmful use of alcohol and a range of mental and behavioral disorders, as well as non-communicable diseases and injuries. Recently, causal relationships have also been established between harmful alcohol consumption and the incidence of infectious diseases, such as tuberculosis and HIV/AIDS. Moreover, alcohol consumption is linked to a higher risk of noncommunicable diseases, such as liver disease, and several cancers, including breast, liver, head and neck, esophageal, and colorectal cancer. In addition to the health consequences, harmful alcohol use leads to significant social and economic losses for individuals and society as a whole [4,5].

Addressing cravings offers a potential alternative for reducing alcohol intake or achieving abstinence. The concept of cravings was first elucidated by Jellinek and colleagues in 1952 as a critical component of the alcohol dependence syndrome [6]. Cravings refer to the subjective urge or intense desire to consume substances and play a central role in driving substance use [7]. Current conceptualizations of cravings incorporate behavioral theories rooted in classical conditioning, as well as cognitive, motivational, and neurobiological factors. At the molecular level, alcohol craving has been linked to persistent dysregulation of dopaminergic, glutamatergic, and GABAergic signaling within mesocorticolimbic circuits, with chronic alcohol exposure inducing neuroadaptations in these pathways that enhance cue reactivity and promote relapse [8]. As per the Diagnostic and Statistical Manual of Mental Disorders (DSM) 5, cravings represent a diagnostic criterion for alcohol dependence syndrome and are defined as the desire to re-experience the effects of previously used psychoactive substances [9]. A committee of addiction specialists convened by the WHO defined cravings as the inclination to seek a repetition of the effects induced by a specific substance. Cravings may manifest during substance consumption, at the onset of abstinence, or after prolonged periods of abstinence, often accompanied by changes in mood, behavior, and cognition [10].

In a review of double-blind, placebo-controlled trials (RCTs) evaluating the effectiveness of medications for controlling craving, it was demonstrated that effective management of intense and often irresistible alcohol cravings can reduce substance use and its associated harms [11]. Specific drugs for the treatment of alcoholism, particularly opioid antagonists like Naltrexone, demonstrate promising outcomes in addressing cravings and supporting abstinence [12,13]. Numerous medications have been explored for treating AUD, encompassing anticonvulsants, psychotropic agents, and anti-smoking medications [14,15].

Despite the availability of diverse pharmacological agents, comprehensive guidelines for effectively managing cravings in AUD are lacking. While clinical trials typically emphasize abstinence as an outcome measure, a subset of studies also assess secondary outcomes, such as cravings [3]. This limited focus may contribute to the paucity of research specifically targeting craving management, underscoring the need for dedicated attention and tailored therapeutic approaches. Furthermore, most pharmacological trials in Alcohol Use Disorder have focused on long-term relapse prevention strategies. However, craving itself is an acute, urgent motivational state that often arises abruptly and is difficult to control. For this reason, the present review specifically targets short-term pharmacological interventions aimed at rapidly reducing cue-induced craving, with the rationale that effective suppression of immediate craving episodes may facilitate abstinence maintenance and reduce the risk of relapse. This review aims to correlate potential treatments using cue-induced studies to identify immediate or short-term (up to approximately three months) reduction in craving.

## 2. Materials and Methods

This review followed the PRISMA Statement for the transparent reporting of systematic reviews and meta-analyses [16]; the completed PRISMA checklist is available in the Supplementary Material (Table S1). This systematic review is registered on the Open Science Framework (OSF) under the following link: https://osf.io/vc6u8 (Accessed on 9 February 2026).

### 2.1. Eligibility

This review included original clinical trials evaluating pharmacological interventions aimed at reducing craving in individuals with alcohol use disorders. Non-original publications—such as reviews, meta-analyses, case reports, discussion articles, and study protocols—were excluded. We also excluded studies that were not randomized, controlled, or double-blind; those published in languages other than English; studies involving animal models; trials that did not include individuals with alcohol abuse or dependence; studies focusing on non-pharmacological interventions for alcohol craving; and investigations assessing alcohol-related outcomes other than craving.

### 2.2. Information Sources

The Pubmed database from the US National Library of Medicine and PsycINFO database were used to identify relevant studies.

### 2.3. Search Strategy

The search was conducted on 13 January 2025, in the PubMed and PsycINFO databases. The keywords used were ‘craving’, ‘alcohol’, and ‘randomized controlled blind’, which were combined with additional synonyms (Supplementary File S1). The filters ‘Clinical Trial’ and ‘Humans’ were applied in both databases.

### 2.4. Study Selection

Study selection was conducted in multiple stages. First, the first and second authors screened the abstracts of all retrieved records, excluding duplicate publications and studies unrelated to alcohol. In the final step, the first author assessed the full texts of the remaining articles and determined eligibility based on the predefined inclusion and exclusion criteria.

### 2.5. Data Collection Process

Study inclusion was conducted independently by the first and second authors. Methodological quality was assessed using the Study Quality Guide developed by the Cochrane Consumers and Communication Review Group [17], evaluating six domains of risk of bias: random sequence generation, allocation concealment, blinding of participants and personnel, blinding of outcome assessment, completeness of outcome data, and selective reporting. When discrepancies arose between the first and second authors regarding study inclusion or data presentation, the last author resolved them and made the final decision.

### 2.6. Data Items

For each included study, data were extracted on the following variables: study title, authorship, year of publication, sample size, mean participant age, sample characteristics, study setting, treatment-seeking status, study design, measures of craving and other relevant outcomes, intervention characteristics, primary and secondary outcomes, and dropout rates.

### 2.7. Inclusion and Exclusion Criteria

Eligible studies were randomized, double-blind, placebo-controlled clinical trials conducted in individuals with alcohol dependence. We excluded studies involving animal subjects, the use of substances other than alcohol, the presence of psychiatric comorbidities, and those that did not evaluate craving as an outcome.

### 2.8. Selection Articles

**Figure 1:**
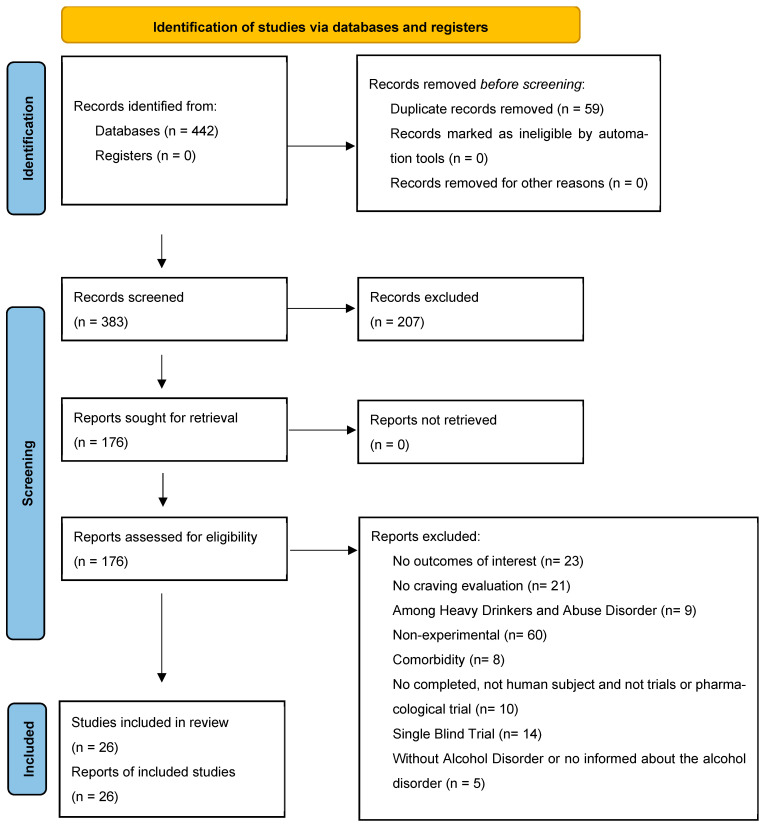
PRISMA flow diagram of study screening and selection.

A total of 442 records were identified through database searches. After removing 59 duplicate records, 383 studies were screened based on titles and abstracts. During this initial screening phase, 207 records that clearly did not meet the eligibility criteria or did not address the research question were excluded. Full texts of 176 reports were subsequently assessed for eligibility. Of these, 150 were excluded for the following reasons: lack of relevant outcomes (n = 23); co craving evaluation (n = 21) studies conducted among heavy drinkers or individuals with alcohol misuse rather than alcohol use disorder (n = 9); non-experimental designs (n = 60); presence of psychiatric comorbidities (n = 8); incomplete studies, non-human subjects, or non-clinical/non-pharmacological trials (n = 10); single-blind trials (n = 14); and studies without a precise diagnosis of alcohol use disorder or insufficient information regarding alcohol dependence (n = 5). The PRISMA flowchart (Figure 1) details records identified, screened, excluded, and studies included.

### 2.9. Qualitative Analysis of Studies

The selected articles were qualitatively assessed using the Joanna Briggs Institute Critical Appraisal Checklist for Randomized Controlled Trials [18], which comprises 13 items evaluating key methodological domains, including randomization procedures, allocation concealment, baseline comparability of groups, blinding of participants, personnel and outcome assessors, completeness of follow-up, adherence to the intention-to-treat principle, consistency and reliability of outcome measurement, appropriateness of statistical analyses, and overall suitability of the trial design.

All 13 quality assessment items were applied to each included study, with responses categorized as “Yes” (1 point), “No” (0 points), “Unclear” (0.5 points), or “Not applicable” (1 point) (Table 1). A score of 10 was defined as the minimum threshold for inclusion. None of the studies scored below this threshold. Only one study did not achieve the maximum score of 13, while all remaining studies attained the highest possible score. The mean quality score across studies was 12.96, indicating that the included trials were of high methodological quality and presented a low overall risk of bias.

## 3. Results

Table 2 summarizes the studies included in the present systematic review. The selected studies exhibited a broad range of foci and sample sizes, with individual trials ranging from 16 to 125 participants (mean = 44), yielding a total of 1097 participants across all studies. Most participants were recruited from outpatient settings and were predominantly male, with ages ranging from 15 to 65 years and a mean age in early adulthood. All participants met established diagnostic criteria for alcohol dependence and heavy drinking, as defined by DSM-III, DSM-IV, the NIAAA AUDIT, and the International Classification of Diseases (ICD-10). The earliest study included in the review was published in 1992, while the most recent was published in 2019, and the majority of studies originated from North America. Specifically, 80.77% (21 studies) are from the US, 15.38% (4 studies) are from Europe and Russia, and 3.84% (1 study) is from Canada.

Regarding measures tools, most studies used the Visual Analog Scale (VAS) (38.4%), followed by the Alcohol Urge Questionnaire (AUQ) (34.6%), the Alcohol Craving Questionnaire (ACQ), and Breath Alcohol Concentration (BrAc) (11.5%). Other scales employed included the Desire for Alcohol Questionnaire (Short DAQ), Tiffany Craving Questionnaire, Alcohol Craving Questionnaire, Yale Craving Scale (YCS), Cue-induced Craving task, Capture measures of SR, Obsessive Compulsive Drinking Scale (OCDS), the Pennsylvania Alcohol Craving Scale (PACS), and self-rating measures of alcohol craving.

The study durations varied from 1 to 98 days, with a mean of 14.2 days per study. The average dropout rate was 2.86%, although dropout rates varied widely, affecting the robustness of the findings. Notably, 42.3% (11 studies) reported no dropouts, while 11.5% (3 studies) did not report dropout data. The study with the highest dropout rate reported a 28% rate. High dropout rates in some studies highlight the challenges of maintaining participant engagement in clinical trials, potentially affecting the generalizability and reliability of results.

In terms of the primary outcome to reduce craving, 15 (57.7%) studies demonstrated positive results compared to placebo, 4 studies (15.38%) showed a non-significant reduction in craving, 6 studies (23%) found no difference compared to placebo, and 2 studies (7.7%) reported an increase in craving compared to placebo. Regarding the promotion of abstinence or reduction in drinking days, this outcome was assessed in 16 (61.5%) studies. Of these, 2 studies reported favorable results for maintaining abstinence in the control group, 7 reported a reduction in the number of drinks in the control group, and 7 found no reduction compared with placebo.

### 3.1. Naltrexone

Naltrexone acts as a competitive antagonist at opioid receptors, thereby inhibiting alcohol-induced dopamine release. This blockade attenuates the reinforcing effects of alcohol, reducing craving and behavioral control loss [3]. Naltrexone was used in experimental cue-induced craving studies in four trials: three as monotherapy [19,20,21] and one trial where it was tested in monotherapy versus placebo and in combination with Ondansetron [22].

In all four studies, naltrexone was associated with a short-term reduction in craving when patients were exposed to situations that induced the desire to drink. The study durations ranged from 4 to 10 days. In the study in which naltrexone was combined with ondansetron, there was a significant reduction in craving compared with placebo. Naltrexone, with or without ondansetron, also decreased alcohol-induced activation of the ventral striatum. Ondansetron alone showed similar results to naltrexone and the combination in the overall analysis, suggesting it may be an effective adjunct in the treatment of craving alongside opioid antagonists [22].

Regarding abstinence or reduction in the number of drinks, naltrexone was evaluated in two studies. One study [20] found it favorable compared to placebo in maintaining abstinence, while another study [21] demonstrated a reduction in the number of drinks per day.

In the studies included in our review, naltrexone was generally well tolerated, with predominantly mild adverse events. In the adolescent sample evaluated by Miranda et al. [21], naltrexone was described as overall well-tolerated, and no specific adverse events or treatment-limiting toxicities were highlighted. In the experimental study by Ray et al. [19], insomnia was the only adverse event reported as more frequent with naltrexone compared with placebo, and no serious adverse events or medication-related discontinuations were observed. Similarly, in the neuroimaging trial conducted by Myrick et al., naltrexone (alone or in combination with ondansetron) was associated with a higher incidence of nausea, vomiting, and dizziness relative to comparator conditions. Still, these events were not considered serious and did not result in systematic withdrawal from the study.

### 3.2. GABAergic Agents

Gamma-Aminobutyric Acid (GABA) is an aminobutyric acid in which the amino group is positioned at the end of the carbon chain. It is the primary inhibitory neurotransmitter in the Central Nervous System (CNS), playing a critical role in regulating neuronal excitability and leading to neurosynaptic inhibition [45]. The inhibitory action of GABA, primarily through activation of the GABA-A receptor, has been associated with aggressive behavior and impulsivity in humans. GABA-A receptors are also the primary site of action for several neuroactive drugs, including benzodiazepines, barbiturates, picrotoxin, and muscimol. In contrast, GABA-B receptors are involved in the actions of baclofen and GHB (Gamma-Hydroxybutyric acid), which are responsible for regulating muscle tone and possibly in pain [45,46].

Acamprosate, a structural derivative of GABA, is thought to modulate alcohol consumption by interacting with calcium channels, enhancing GABAergic transmission, and attenuating glutamate activity at NMDA receptors. These pharmacological actions are postulated to diminish positive reinforcement associated with alcohol consumption and mitigate cravings during withdrawal [47]. In 1 double-blind, placebo-controlled study, acamprosate effectively reduced craving over a 21-day evaluation period when craving was induced by alcohol cues involving visual, tactile, olfactory, and auditory stimuli. While the study did not demonstrate significant effects on abstinence, there was a trend indicating a reduction in heavy drinking days, supporting acamprosate’s potential role in promoting abstinence. In that trial, acamprosate was administered at 1998 mg/day for 21 days and was generally well tolerated, with no serious adverse events, no treatment discontinuations attributable to the medication, and no consistent pattern of specific side effects reported [23].

Memantine, a medication typically used in Alzheimer’s treatment, belongs to the class of N-methyl-D-aspartate (NMDA) receptor antagonists and is not strictly a GABAergic agent. However, by inhibiting glutamatergic receptors, it exerts indirect GABAergic effects [48]. Memantine, known for its role in slowing the progression of dementia syndromes, was evaluated in 2 studies. In one study, memantine reduced craving compared to placebo [24]; in the other study [25], pretreatment with memantine reduced alcohol craving before, but not after, alcohol administration. Memantine was noted to increase the dissociative effects of alcohol without altering its sedative effect [25].

### 3.3. Anticonvulsant Agents

Anticonvulsant agents have been primarily evaluated as an alternative to benzodiazepines in the management of alcohol withdrawal and for continuous outpatient care for alcohol dependence, aimed at achieving either complete abstinence or harm reduction [49].

Topiramate was initially developed as an antidiabetic compound and later introduced into clinical practice as an anticonvulsant, with a chemical structure related to that of acetazolamide [50,51]. Its mechanism of action involves positive allosteric modulation of GABA A receptors, facilitating chloride ion entry into neurons and enhancing inhibitory GABAergic neurotransmission. This effect is achieved by binding to sites on GABA A receptors distinct from those targeted by benzodiazepines [52]. In a 5-week experimental study, topiramate was administered at a dose of 200 mg/day and demonstrated significant reductions in alcohol craving and consumption compared to placebo and aripiprazole [26].

Gabapentin has been investigated for its potential in reducing alcohol consumption and craving. However, in its sole experimental study, gabapentin did not demonstrate significant differences compared to placebo in reducing consumption and craving, though it was confirmed to be a very safe medication [27].

### 3.4. Varenicline

Varenicline is a partial agonist of nicotinic acetylcholine receptors, particularly blocking activation of α4β2 receptors and subsequently stimulating the central mesolimbic dopamine system, a pathway implicated in reinforcement and reward associated with smoking [53]. Varenicline, widely used in smoking cessation, has been evaluated for its effects on alcohol consumption [54,55]. In a study by Roberts (2017), patients treated with varenicline reported a significant reduction in craving and fewer drinks consumed than those receiving a placebo [28]. Two additional studies further supported these findings, demonstrating that varenicline at a dose of 2 mg per day led to a reduction in daily alcohol intake and cravings compared to placebo [29,30].

### 3.5. Other Psychotropic Drugs

Due to the limited availability of specific medications for the treatment of AUD, various drugs traditionally used for mental health disorders have been explored over the years. For short-term craving reduction, the following drugs have been tested: Aripiprazole, Citalopram, Haloperidol, Ketamine, Naloxone, and Quetiapine.

Relating to antipsychotics, aripiprazole was evaluated in 2 RCTs, with one study showing a reduction in the number of drinks consumed [31]. In contrast, two studies found no significant difference in the attenuation of craving compared with placebo [26,31]. Quetiapine, another secondary antipsychotic, and haloperidol, a first-generation butyrophenone-class antipsychotic, both showed reductions in craving. Still, they were not effective in maintaining abstinence or reducing the overall number of drinks [31,32,33].

Citalopram, a selective serotonin reuptake inhibitor (SSRI) antidepressant, showed a significant reduction in craving and maintained abstinence compared to placebo in a 4-week study [34]. Ketamine, an NMDA glutamate receptor antagonist, did not show significant reductions in craving, number of drinks, or rates of abstinence compared to placebo [35].

Naloxone, an opioid antagonist, was evaluated solely for its ability to reduce craving compared to a placebo, but was not successful in showing significant effects [36].

### 3.6. Other Drugs

Several other medications, not commonly used for mental or substance use disorders, have been evaluated for their effects on AUD. In total, 6 different drugs were tested, demonstrating varied outcomes. In a 7-day RCT comparing intranasal oxytocin with placebo, a reduction in craving was observed in the oxytocin group, although maintenance of abstinence was not assessed [37]. Three placebo-controlled experimental studies examined the role of appetite-regulating pathways in alcoholism using intravenous ghrelin. In 2 studies, a ghrelin infusion was found to reduce serum leptin levels, a mediator that typically reduces appetite, and these lower levels were negatively correlated with alcohol craving [38,39]. Haass-Koffler (2016) explored the correlation between ghrelin infusion and serum insulin levels but found no significant association between changes in insulin levels and changes in alcohol craving [40].

Other studies attempted to associate a rapid and significant decline in plasma free tryptophan (via ingestion of an amino acid drink lacking tryptophan) with increased craving. In 2 RCTs, no difference was observed compared to placebo in maintaining abstinence, reducing the number of drinks, or reducing craving [41,42].

D-cycloserine has been known for its ability to reduce craving, but did not differ from placebo [43]. Minocycline also showed no significant effect on craving or the number of drinks consumed [44].

Ondansetron, an antiemetic used off-label for AUD, was evaluated alone or in combination with naltrexone for its effects on craving. Using brain blood oxygen level-dependent (BOLD) magnetic resonance imaging as a measure, ondansetron alone showed effects similar to those of naltrexone and the combination therapy in the overall analyses, but it was intermediate in the region-specific analysis [22].

## 4. Discussion

This systematic review synthesizes randomized, double-blind, placebo-controlled experimental trials assessing pharmacological interventions for short-term craving reduction in Alcohol Use Disorder (AUD). By focusing on cue-induced paradigms, it highlights medications that produce rapid-onset anti-craving effects—a therapeutic dimension often overlooked in traditional long-term treatment approaches. Among the 26 studies included, Naltrexone and Varenicline emerged as the most consistently effective agents, with additional, though less robust, evidence supporting Memantine, Acamprosate, and Topiramate.

The convergence of findings around opioid, glutamatergic, and nicotinic modulation reinforces the central role of mesolimbic dopamine regulation in craving suppression. Naltrexone, a μ-opioid receptor antagonist [56], remains the gold standard due to its capacity to attenuate cue-induced dopaminergic activation in the ventral striatum, effectively reducing craving intensity and relapse risk. Its combination with Ondansetron, a 5-HT3 receptor antagonist [57], suggests synergistic modulation of serotonergic and dopaminergic pathways, which could potentiate early control of craving.

Varenicline, a partial agonist of the α4β2 nicotinic acetylcholine receptor [58], also demonstrated rapid reductions in craving and alcohol intake. Beyond its established role in nicotine dependence, its dopaminergic stabilization effects may account for its efficacy in attenuating the salience of alcohol cues. These results are particularly relevant for dual users of alcohol and tobacco, representing an essential intersection of addiction pharmacotherapy.

GABAergic and glutamatergic agents also showed promise for modulating craving. Acamprosate, which normalizes glutamatergic hyperactivity during withdrawal [59], reduced cue-induced craving and drinking behavior within three weeks of treatment. Similarly, Memantine, an NMDA receptor antagonist [60], decreased craving intensity, possibly by restoring the excitatory-inhibitory balance in cortico-limbic circuits. However, its effects on abstinence were not consistently evaluated. Topiramate, with combined GABA-enhancing and glutamate-inhibiting properties [61], showed significant benefits across craving and drinking outcomes, supporting its potential as an acute anti-craving agent. Conversely, Gabapentin, despite well-documented long-term benefits for relapse prevention [62], demonstrated limited short-term efficacy in experimental paradigms. This discrepancy highlights the temporal specificity of pharmacodynamic effects and suggests that the drug’s anxiolytic and sleep-regulating properties may contribute more to sustained rather than immediate craving suppression.

Among psychotropic drugs, Citalopram yielded reductions in craving and enhanced abstinence maintenance, likely through serotonergic modulation of reward sensitivity and impulsivity [63]. Aripiprazole and Quetiapine, by contrast, displayed inconsistent results, possibly reflecting heterogeneous dopaminergic effects across individuals and differing motivational states. Ketamine and Haloperidol were largely ineffective in short-term craving control, underscoring the limited translational utility of agents with nonspecific central nervous system actions in acute craving modulation.

Emerging interest in neuropeptide and hormonal targets such as Oxytocin and Ghrelin opens new avenues for craving modulation. Intranasal oxytocin was associated with reduced cue-induced craving, potentially via attenuation of stress reactivity and enhancement of social cognition [64]. Conversely, ghrelin administration paradoxically increased craving, confirming its role as a pro-appetitive signal in alcohol-seeking behavior [65]. These contrasting outcomes highlight the need for further mechanistic studies exploring neuroendocrine contributions to alcohol motivation.

From a clinical standpoint, the choice of an initial pharmacological agent for alcohol craving should integrate the experimental evidence summarized in this review with current treatment guidelines for AUD. In line with our findings, naltrexone and varenicline can be considered first options for short-term craving reduction. At the same time, broader clinical guidelines identify naltrexone and acamprosate as preferred first-line agents for patients with moderate-to-severe AUD who aim either to reduce heavy drinking or to achieve abstinence [59]. Disulfiram, although approved for AUD, was not evaluated in the trials included in this review and is generally not recommended as an initial anti-craving strategy because it acts primarily through an aversive mechanism, requires strict abstinence and close supervision, and is limited by safety concerns, particularly hepatotoxicity [66]. In clinical practice, short-term pharmacotherapy may be used either to reduce alcohol consumption (for example, by decreasing the number of heavy-drinking days) or to support the initiation and consolidation of abstinence; naltrexone and topiramate have the most substantial evidence for reducing heavy drinking, whereas acamprosate is more consistently associated with maintenance of abstinence. The choice between agents should also take into account medical comorbidities: in significant liver disease, acamprosate is generally preferred, whereas disulfiram and naltrexone should be avoided or used with great caution in patients with acute hepatitis or hepatic failure; in severe renal impairment, acamprosate is contraindicated and drugs such as topiramate require dose adjustment; in individuals with epilepsy, medications with anticonvulsant properties (such as topiramate or gabapentin) may be advantageous; and in pregnancy, pharmacotherapy is usually avoided, with psychosocial interventions remaining the mainstay of care [67]. Pharmacological treatment is best supported in moderate-to-severe AUD. Still, it may be considered in mild AUD when psychosocial approaches alone are insufficient, with careful dose titration and closer monitoring in older adults [68]. When the initial agent yields an inadequate response, options include optimizing adherence and dose within recommended limits, switching to a drug with a different mechanism of action, and, in selected cases, combining medications, always within a structured psychosocial treatment plan.

### Clinical Implications and Future Directions

The identification of fast-acting pharmacotherapies holds meaningful implications for clinical practice. Acute craving surges frequently precipitate relapse during early recovery phases or after detoxification. Agents such as Naltrexone, Varenicline, Acamprosate, and Memantine may be strategically deployed in these high-risk windows to stabilize neurobiological dysregulation and prevent lapse-to-relapse transitions. This short-term approach could complement long-term maintenance therapies, behavioral interventions, or contingency management strategies. Notably, the reviewed studies also highlight methodological limitations that constrain the generalizability of findings. Sample sizes were small, follow-up periods were brief, and craving induction paradigms were heterogeneous. Furthermore, most participants were male and from North American settings, limiting cross-cultural applicability. Standardization of craving induction and measurement (e.g., harmonizing VAS, AUQ, and ACQ instruments) will be essential for comparative and meta-analytic synthesis.

In addition to these evidence-related issues, the present review also has limitations that should be acknowledged. First, we did not perform a quantitative meta-analytic synthesis because the number of eligible studies and the heterogeneity of designs and outcome measures were insufficient to support a meaningful pooled analysis. Second, because our review is based on published trials, the findings may be influenced by publication bias, with negative or null results likely underreported in the available literature. Third, although the JBI appraisal indicated generally high methodological quality across studies, residual sources of bias—such as selective reporting, publication bias, and limited sample sizes—cannot be entirely excluded.

From a translational perspective, future research should prioritize multi-site, adequately powered RCTs that integrate neuroimaging biomarkers, such as BOLD-fMRI, or PET-based receptor occupancy studies, to elucidate mechanisms underlying rapid craving suppression. Combination strategies (e.g., Naltrexone + Ondansetron, or Varenicline + Acamprosate) merit systematic evaluation, as do potential adjunctive uses in comorbid tobacco or anxiety disorders.

## 5. Conclusions

This review positions short-term anti-craving pharmacotherapies as an emerging frontier in AUD management. Naltrexone and Varenicline remain the most evidence-supported options for immediate craving suppression, while Memantine, Acamprosate, and Topiramate show promising but preliminary benefits. Agents such as Oxytocin and Citalopram illustrate the expanding neurobiological diversity of anti-craving mechanisms. Future studies should adopt standardized experimental paradigms, incorporate translational biomarkers, and explore combined pharmacological-behavioral models. These advances will be critical for developing rapid-response treatment algorithms capable of addressing the fluctuating nature of alcohol craving and relapse risk in clinical settings.

## Figures and Tables

**Table 1:** Quality assessment following the JBI criteria for RCT (Scoring criteria—1 = Yes; 0 = No; 0.5 = Unclear; “Not applicable” items were excluded from the total score).

Article	Q1	Q2	Q3	Q4	Q5	Q6	Q7	Q8	Q9	Q10	Q11	Q12	Q13	Total
Ray, 2012 [19]	1	1	1	1	1	1	1	1	1	1	1	1	1	13
Green, 2019 [20]	1	1	1	1	1	1	1	1	1	1	1	1	1	13
Miranda, 2014 [21]	1	1	1	1	1	1	1	1	1	1	1	1	1	13
Myrick, 2009 [22]	1	1	1	1	1	1	1	1	1	1	1	1	1	13
Hammarber, 2009 [23]	1	1	1	1	1	1	1	1	1	1	1	1	1	13
Krupitsky, 2007 [24]	1	1	1	1	1	1	1	0.5	0.5	1	1	1	1	12
Bisaga, 2004 [25]	1	1	1	1	1	1	1	1	1	1	1	1	1	13
Haass-Koffler, 2018 [26]	1	1	1	1	1	1	1	1	1	1	1	1	1	13
Myrick, 2007 [27]	1	1	1	1	1	1	1	1	1	1	1	1	1	13
Roberts, 2017 [28]	1	1	1	1	1	1	1	1	1	1	1	1	1	13
Verplaetse, 2016 [29]	1	1	1	1	1	1	1	1	1	1	1	1	1	13
McKee, 2009 [30]	1	1	1	1	1	1	1	1	1	1	1	1	1	13
Voronin, 2008 [31]	1	1	1	1	1	1	1	1	1	1	1	1	1	13
Ray, 2011 [32]	1	1	1	1	1	1	1	1	1	1	1	1	1	13
Modell, 1993 [33]	1	1	1	1	1	1	1	1	1	1	1	1	1	13
Naranjo, 1992 [34]	1	1	1	1	1	1	1	1	1	1	1	1	1	13
Krystal, 1998 [35]	1	1	1	1	1	1	1	1	1	1	1	1	1	13
Lieb, 2013 [36]	1	1	1	1	1	1	1	1	1	1	1	1	1	13
Mitchell, 2016 [37]	1	1	1	1	1	1	1	1	1	1	1	1	1	13
Haass-Koffler, 2015 [38]	1	1	1	1	1	1	1	1	1	1	1	1	1	13
Leggio, 2014 [39]	1	1	1	1	1	1	1	1	1	1	1	1	1	13
Haass-Koffler, 2016 [40]	1	1	1	1	1	1	1	1	1	1	1	1	1	13
Petrakis, 2002 [41]	1	1	1	1	1	1	1	1	1	1	1	1	1	13
Petrakis, 2001 [42]	1	1	1	1	1	1	1	1	1	1	1	1	1	13
Watson, 2011 [43]	1	1	1	1	1	1	1	1	1	1	1	1	1	13
Petrakis, 2019 [44]	1	1	1	1	1	1	1	1	1	1	1	1	1	13

**Table 2:** Main information of the included studies.

Author, Year, Country	Sample: n; Age (SD or Range); % Male; Alcohol Use Pattern; Setting	Measures (Alcohol Use Pattern and Craving)	Intervention	Follow-Up	Outcomes (Abstinence and/or Craving)	Dropout
Bisaga, E., et al. (2004) [25]; USA	88; 27.9; 66.6% male; heavy drinkers and alcohol dependence; inpatients	ACS and VAS; BAES	Memantine	2 weeks	Craving significantly reduced vs. placebo	N/A
Green, R.J., et al. (2019) [20]; USA	87; 26.8 ± 6.15; 66.7% male; heavy drinkers; outpatient	AUDIT; BAES and AUD	Naltrexone (50 mg/day)	5 days	Craving reduced vs. placebo; number of drinks reduced in the control group	11.5%
Haass-Koffler, C.L., et al. (2018) [26]; USA	90; 40.6 ± 11.9; 63.3% male; heavy drinkers and alcohol dependence; outpatient	DSM 4; AUQ and BrAC	Topiramate; Aripiprazole	5 weeks	Topiramate reduced craving and drinking vs. placebo; aripiprazole showed no difference	10.0%
Haass-Koffler, C.L., et al. (2015) [38]; USA	45; N/A; N/A; alcohol abuse and dependence; outpatient	DSM 4; VAS or a Juice VAS	Ghrelin IV	1 day	Craving increased vs. placebo	4.4%
Haass-Koffler, C.L., et al. (2016) [40]; USA	43; N/A; N/A; alcohol dependence and heavy drinkers; outpatient	DSM 4; NIAAA; VAS or Juice VAS	Ghrelin IV	1 day	Non-significant reduction in craving vs. placebo	N/A
Hammarberg, A., et al. (2009) [23]; Sweden	56; acamprosate: 50.2 ± 7.6, placebo: 49.8 ± 7.3; 53.5% male; alcohol dependence; outpatient	DSM 4; Swedish version of the Desire for Alcohol Questionnaire; VAS and Alcohol cues consisted of a mixture of alcohol related visual, tactile, olfactory, and auditory stimuli.	Acamprosate (1998 mg/day)	21 days	Craving reduced vs. placebo; number of drinks reduced in the control group	25%
Krupitsky, E.M., et al. (2007) [24]; Russia	38; 39.2 ± 9.0; 100% male; alcohol dependence; inpatients	ICD-10; DSM 4; VAS	Memantine	3 days	Craving significantly reduced vs. placebo	0%
Krystal, J.H., et al. (1998) [35]; USA	20; 44 ± 10.5; 100% male; alcohol dependence; inpatients	DSM-3; VAS	Ketamine	3 days	No difference in abstinence vs. placebo; non-significant reduction in craving	0%
Leggio, L., et al. (2014) [39]; USA	45; 44.7 ± 9.1; 20% male; alcohol dependence; outpatient	VAS: The Alcohol Attention Scale and exposed to neutral and alcohol cues.	Ghrelin IV	1 day	Craving increased vs. placebo	0%
Lieb, M., et al. (2013 [36]); Germany	20; N/A; 100% male; alcohol dependence; inpatients	DSM 4; ACQ	Naloxone	2 days	No difference in craving vs. placebo	0%
McKee, S.A., et al. (2009) [30]; USA	20; varenicline: 34.20 ± 12.08, placebo: 35.30 ± 12.71; 80% male; heavy drinkers; N/A	DSM 4; AUQ	Varenicline	7 days	Craving reduced vs. placebo; number of drinks reduced in the control group	0%
Miranda, R., et al. (2014) [21]; USA	22; 18.3 ± 0.95; 45% male; heavy drinkers; outpatient	DSM 4 and AUQ	Naltrexone (50 mg/day)	8–10 days	Abstinence favored in control group; craving reduced vs. placebo	0%
Mitchell, J.M., et al. (2016) [37]; USA	32; 28.9 ± 7.15; 59.3% male; alcohol abuse; outpatient	DSM 4; AUDIT; AUQ; 7-point Likert scale and Cue-induced Craving Task	Intranasal Oxytocin	7 days	Craving significantly reduced vs. placebo	0%
Modell, J.G., et al., (1993) [33]; USA	16; 39; 62.5% male; alcohol dependence; outpatient	DSM-3; Self-ratings of alcohol craving	Haloperidol	14 weeks	No difference in abstinence vs. placebo; craving reduced	N/A
Myrick, H., et al. (2008) [22]; USA	125; naltrexone: 27.22 ± 8.84, ondansetron: 25.13 ± 6.88, combination: 26.15 ± 8.82, placebo: 24.75 ± 5.74, control: 25.18 ± 4.00; 72.9% male; alcohol dependence; outpatient	DSM 4; CIWA-Ar; Self-ratings of alcohol craving	Naltrexone; Ondansetron; Naltrexone (50 mg/day) + Ondansetron	7 days	Craving significantly reduced vs. placebo	28%
Myrick, H., et al. (2007) [27]; USA	35; gabapentin: 34.8 ± 14.1, placebo: 32.3 ± 10.1; 94.2% male; alcohol dependence; outpatient	DSM-5; SCID and ACQ	Gabapentin	8 days	No difference in abstinence or craving vs. placebo	0%
Naranjo, C.A., et al. (1992) [34]; Canada	16; 38.5 ± 13.2; 81% male; heavy drinkers; outpatient	Self-ratings of alcohol craving	Citalopram	4 weeks	Abstinence favored in control group; craving reduced vs. placebo	5%
Petrakis, I.L., et al. (2001) [42]; USA	16; 43.1 ± 6.3; 94% male; alcohol dependence; inpatients	DSM-3 and Tiffany Craving Questionnaire	Active tryptophan depletion	1 week	No difference in abstinence or craving vs. placebo	0%
Petrakis, I.L., et al. (2002) [41]; USA	12; 27.7 ± 9.2; 83.3% male; alcohol abuse and dependence; outpatient	DSM 4; CIWA-Ar; TLFB and Addiction Severity Index (ASI); Tiffany Craving Questionnaire	Active tryptophan depletion	1 week	No difference in abstinence or craving vs. placebo	10.0%
Petrakis, I.L., et al. (2019) [44]; USA	49; 34.4 ± 10.3; 71.4% male; Alcohol Use Disorder; outpatient	AUDIT; SCID and Yale Craving Scale	Minocycline	10 days	No difference in abstinence or craving vs. placebo	2%
Ray, L.A., et al. (2011) [32]; USA	20; placebo: 34.1 ± 11.74, quetiapine: 34.5 ± 14.21; 80% male; alcohol dependence; outpatient	DSM 4; PACS; OCDS; AUQ and Alcohol administration cue-exposure	Quetiapine	6 weeks	No difference in abstinence vs. placebo; craving reduced	25%
Ray, L.A., et al. (2012) [19]; USA	35; 22.3 ± 1.98; 71% male; heavy drinkers; outpatient	AUDIT; AUQ and Intravenous alcohol administration	Naltrexone (25–50 mg/day)	4 days	Craving reduced vs. placebo; number of drinks reduced in the control group	8.5%
Roberts, W., et al. (2017) [28]; USA	77; placebo: 32.61 ± 10.21, varenicline: 34.85 ± 11.17; 65% male; heavy drinkers (smokers and non-smokers); outpatient	DSM 4; AUDIT and VAS	Varenicline	10 days	Craving reduced vs. placebo; number of drinks reduced in the control group	0%
Verplaetse, T.L., et al. (2016) [29]; USA	60; placebo: 34.2 ± 9.52, varenicline 1 mg: 33.35 ± 8.51, varenicline 2 mg: 34.15 ± 11.6; 68% male; heavy drinkers and alcohol dependence; outpatient	DSM 4; Tiffany Questionnaire of Smoking Urges (QSU-Brief); AES; AUQ and VAS	Varenicline	10 days	Craving reduced vs. placebo; number of drinks reduced in the control group	14.2%
Voronin, K., et al. (2008) [31]; USA	30; aripiprazole: 29.0 ± 10.6, placebo: 25.5 ± 8.9; 83.3% male; alcohol dependence; outpatient	DSM 4; OCDS; SAAST; ADS and ACQ;	Aripiprazole	8 days	Number of drinks reduced in the control group; non-significant reduction in craving	0%
Watson, B.J., et al. (2011) [43]; UK	16; D-cycloserine: 45.2 ± 10.1, placebo: 43.9 ± 8.5; 71.4% male; alcohol dependence; outpatient	DSM 4; ACDS; AUQ, VAS, and Cue-exposure	D-cycloserine	3 weeks	No difference in craving vs. placebo	12.5%

## Data Availability

No new data were created or analyzed in this study. Data sharing is not applicable to this article.

## Supplementary Materials

The following supporting information can be downloaded at: https://www.mdpi.com/article/10.3390/medicines13010007/s1, Table S1: PRISMA 2020 Checklist for Reporting of This Systematic Review, Including Itemized Compliance and Manuscript Location; Supplementary File S1: Full Database-Specific Search Strategies for PubMed (MEDLINE) and PsycINFO (APA PsycNET/EBSCOhost).
