# Supplementary material for: Pharmacotherapy for Alcohol Craving Reduction: Efficacy of Short-Term Treatments in Alcohol Use Disorder
Source: Medicines (Basel). 2026 Feb 14;13(1):7. doi: 10.3390/medicines13010007 (PMC12921795; doi:10.3390/medicines13010007)
Supplement: Supplementary file 1 [file medicines-13-00007-s001.zip › Supplementary File S1.pdf]

Supplementary File 1. Full Database-Specific Search Strategies for PubMed (MEDLINE) and PsycINFO (APA PsycNET/EBSCOhost).

Full Database-Specific Search Strategies

Databases

- PubMed (MEDLINE)
- PsycINFO (APA PsycNET / EBSCOhost)

Date of search

January 13, 2025

1. PubMed Search Strategy

The PubMed search combined free-text terms and controlled vocabulary (MeSH) related to alcohol craving and randomized controlled trials. The following search string was used:

```
(
  craving[Title/Abstract]
  OR cravings[Title/Abstract]
  OR "cue-induced craving"[Title/Abstract]
  OR "alcohol craving"[Title/Abstract]
)
AND
(
  alcohol[Title/Abstract]
  OR alcoholism[Title/Abstract]
  OR "alcohol use disorder"[Title/Abstract]
  OR "alcohol dependence"[Title/Abstract]
  OR ethanol[Title/Abstract]
)
AND
(
  randomized controlled trial[Publication Type]
  OR "randomized controlled trial"[Title/Abstract]
  OR randomized[Title/Abstract]
  OR placebo[Title/Abstract]
  OR double-blind[Title/Abstract]
  OR "double blind"[Title/Abstract]
)
AND
(
  Humans[MeSH]
)
```

Filters applied:

- Article type: Clinical Trial
- Species: Humans

2. PsycINFO Search Strategy

The PsycINFO search combined title and abstract keywords with APA Thesaurus descriptors. The following search strategy was applied:

```
(
  TI(craving OR cravings OR "cue-induced craving" OR "alcohol craving")
  OR
  AB(craving OR cravings OR "cue-induced craving" OR "alcohol craving")
)
AND
(
  TI(alcohol OR alcoholism OR "alcohol use disorder" OR "alcohol dependence")
  OR
  AB(alcohol OR alcoholism OR "alcohol use disorder" OR "alcohol dependence")
)
AND
(
  DE "Alcohol Drinking"
  OR DE "Alcoholism"
)
AND
(
  TI(randomized OR placebo OR "double blind")
  OR
  AB(randomized OR placebo OR "double blind")
)
AND
(
  DE "Clinical Trials"
  OR DE "Treatment Outcome"
)
```

Limits applied:

- Methodology: Clinical Trial
- Population: Human
